# Research Progress on the Regulatory Mechanisms of Gut Microbiota in Methamphetamine Addiction and Targeted Interventions

**DOI:** 10.1111/adb.70171

**Published:** 2026-09-08

**Authors:** Jihao Yang, Yishuang Dai, Jia Li

**Affiliations:** ^1^ School of Acupuncture and Tuina Guizhou University of Traditional Chinese Medicine Guiyang China; ^2^ Chongqing Medical and Pharmaceutical College Chongqing China

**Keywords:** gut–brain axis, gut microbiota, methamphetamine addiction, neuroinflammation, targeted interventions

## Abstract

Methamphetamine (METH) is a globally prevalent, highly addictive synthetic psychostimulant, for which no FDA‐approved pharmacotherapy is currently available for METH use disorder (MUD). The microbiota–gut–brain axis has been well established as a key regulatory pathway in substance use disorders, yet its specific mechanistic basis and translational potential in METH addiction remain to be systematically elucidated. This review synthesizes current preclinical and clinical evidence demonstrating that METH exposure induces profound gut microbiota dysbiosis, characterized by the depletion of beneficial genera such as *Faecalibacterium* and *Lactobacillus*, enrichment of proinflammatory phylum Proteobacteria and concurrent dysregulation of microbial metabolites including short‐chain fatty acids (SCFAs), tryptophan derivatives and bile acids. These microbial signals mediate bidirectional gut–CNS crosstalk through neuroimmune, neuroendocrine (hypothalamic–pituitary–adrenal (HPA) axis) and vagal pathways, thereby exacerbating the core central pathologies of METH addiction: neurotransmitter system imbalance, neuroinflammation and oxidative stress and dysfunction of addiction‐related neural circuits. We further elaborate that gut microbiota–driven epigenetic modifications and transgenerational effects reinforce the persistence and heritability of addictive phenotypes. Importantly, microbiota‐targeted interventions, including probiotics, prebiotics, faecal microbiota transplantation (FMT) and dietary modulation, can alleviate METH‐induced affective disturbances (anxiety/depression‐like behaviours), multiorgan damage (neurotoxicity, reproductive impairment) and relapse risk, via restoring gut microbial homeostasis, repairing intestinal barrier integrity and normalizing gut–brain axis signalling. Collectively, this review positions the gut microbiota as a critical peripheral regulatory node in METH addiction, providing a robust preclinical foundation for the development of gut–brain axis–targeted combination therapies for MUD.

## Abstract 2

METH induces gut dysbiosis with increased proinflammatory and decreased beneficial bacteria, impairs mucosal barrier with reduced SCFAs/Occludin and increased permeability, leading to endotoxin leakage. This activates HPA axis, vagus‐CNS pathways, oxidative stress, and gut‐testis axis disturbance, all synergistically causing neurotoxicity, addiction, and multisystem pathology.

## 1 Introduction

Previous studies have shown that addictive substances including alcohol, morphine and methadone trigger significant gut microbiota dysbiosis, which shares core features with the microbial alterations observed in METH exposure, such as depletion of beneficial commensal bacteria, enrichment of proinflammatory pathogens and impaired intestinal barrier function [1]. Dysregulated microbiota modulates the gut–brain axis via metabolites including SCFAs and neurotransmitter precursors, driving neuroinflammation and maladaptive reward circuitry to form a vicious cycle of addiction [1]. Extracellular vesicles derived from gut microbiota have also been identified as mediators of alcohol addiction‐related behaviours [2]. The gut–brain axis is the core bidirectional communication pathway between the gastrointestinal tract and the central nervous system (CNS). Gut microbiota–derived metabolites, such as tryptophan and tyrosine derivatives, regulate addiction‐related neurotransmitter balance, microglial activation and dopamine reward signal transduction via neuroimmunomodulation [3, 4, 5], providing a critical peripheral regulatory perspective for addiction mechanism research.

Among addictive substances, METH is one of the most widely abused synthetic psychostimulants worldwide, with a growing epidemic and severe treatment gaps. Initially developed for the treatment of attention deficit hyperactivity disorder (ADHD) and short‐term weight management, METH has become a prevalent illicit drug, with global usage and associated mortality rising sharply since the mid‐2010s [6, 7, 8]. METH use imposes a disproportionate burden on vulnerable populations, including Australian adolescents and men who have sex with men in the United Kingdom, and is frequently comorbid with behavioural disorders such as conduct disorder [9, 10]. Despite decades of research, no pharmacological therapy for MUD has been approved by the US Food and Drug Administration (FDA), and effective treatments remain scarce globally [11, 12, 13, 14]. Current clinical management mainly relies on psychosocial interventions including withdrawal support and contingency management, with limited long‐term efficacy [15].

The gut microbiota is a complex community of trillions of microorganisms colonizing the gastrointestinal tract [16, 17], which maintains host homeostasis via regulating metabolism, immune function and neural activity, and is a core component of the gut–brain axis [17]. Gut microbiota mediates bidirectional gut–CNS communication through metabolites such as SCFAs and neurotransmitters [1, 18], directly modulating brain reward circuitry, addictive substance metabolism and associated behaviours. To date, most METH toxicity research has focused on the CNS, circulatory and respiratory systems, with limited attention to the gastrointestinal tract. As the ‘second brain’ and the body's largest immune organ, the gastrointestinal tract is highly sensitive to METH: Preclinical studies have confirmed that METH exposure reduces gut microbial diversity in mice, with increased pathogenic bacteria and decreased beneficial commensals [19], and these microbial alterations are tightly linked to METH‐induced depressive‐like behaviours and neurotoxicity [20]. Compelling clinical evidence has further identified characteristic gut microbiota dysbiosis in MUD patients, which correlates with psychiatric symptoms and impulsivity [21, 22]. In recent years, the critical role of gut microbiota and its metabolites (especially tryptophan derivatives) in regulating host behaviour and brain function has received extensive attention, providing a key entry point for dissecting the peripheral–central regulatory network of METH addiction, and serving as the core logical starting point for this review to systematically explore the mechanisms and intervention strategies of METH addiction.

In view of this, this review focuses on globally prevalent METH addiction, for which no FDA‐approved pharmacotherapies are available. We systematically explore the underlying regulatory mechanisms, clarify how METH exposure‐induced gut microbiota dysbiosis (depletion of beneficial bacteria, enrichment of proinflammatory bacteria and dysregulation of key microbial metabolites including SCFAs and tryptophan derivatives) exacerbates central pathologies such as neurotransmitter imbalance and neuroinflammation via gut–brain axis communication mediated by neuroimmune, neuroendocrine and vagal pathways. We further analyse the microbiota‐driven epigenetic modifications and transgenerational effects of METH addiction, verify the alleviating effects of microbiota‐targeted interventions (e.g., probiotics) on METH‐related pathological abnormalities, identify the gut microbiota as a core peripheral regulatory node of METH addiction and provide a theoretical basis for the development of gut–brain axis–targeted therapies for MUD.

## 2 Core CNS Pathological Targets of METH Addiction Modulated by Gut Microbiota

**FIGURE 1:**
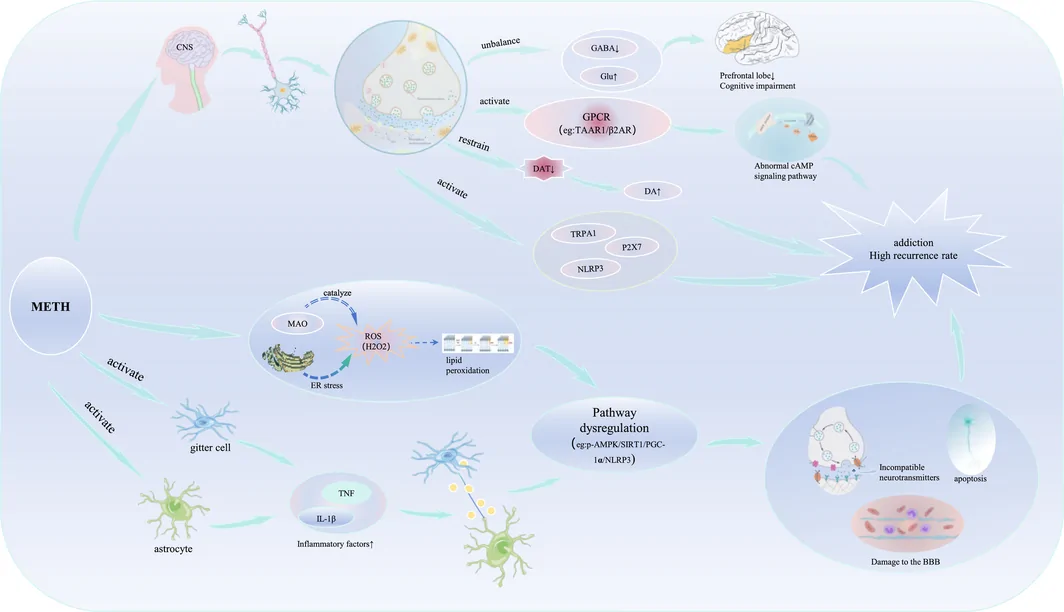
The regulatory mechanisms of METH on CNS and related pathological processes. METH can act on multiple components in the CNS, including neurons, microglia and astrocytes: It alters the levels of neurotransmitters (GABA, Glu, DA) and dopamine transporter (DAT) function, induces ROS production, ER stress and lipid peroxidation via MAO, activates proinflammatory factors (TNF‐α, IL‐1β) and NLRP3 inflammasome signalling and causes dysregulation of metabolic pathways (e.g., AMPK/SIRT1/PGC‐1α).

The core pathological hallmarks of METH addiction in the CNS include neurotransmitter system imbalance, synergistic damage of neuroinflammation and oxidative stress and dysfunction of addiction‐related neural circuits. Gut microbiota and its metabolites are the key peripheral regulators that directly modulate these CNS pathological processes, forming a bidirectional pathological cascade between the gut and the brain (Figure 1).

### 2.1 Regulatory Mechanisms of Neurotransmitters and Receptors

METH drives the addictive reward effect primarily by inhibiting the DAT to increase synaptic dopamine (DA) levels [23, 24], while dysregulating glutamatergic and GABAergic transmission to induce cognitive deficits. Gut microbiota–derived metabolites directly modulate this core reward pathway: microbiota‐derived SCFAs regulate the expression of dopamine synthetase in the nucleus accumbens (NAc) to alter DA release [25], while microbiota‐derived tryptophan metabolites modulate glutamatergic synaptic transmission via the aryl hydrocarbon receptor (AhR) [26], thereby shaping METH‐induced reward and cognitive phenotypes.

Long‐term METH exposure leads to persistent disorders of the neurotransmitter system, including mitochondrial dysfunction [23], synaptic plasticity damage [26, 27] and synergistic dysregulation of dopaminergic, glutamatergic and immune pathways [28]. These changes jointly maintain the persistence and high relapse rate of addiction. Notably, current studies have not yet clarified the molecular details of this synergistic dysregulation, nor have they fully explored its time‐dependent changes, lacking direct causal evidence for the prevention or reversal of these pathological changes via microbiota‐targeted modulation.

### 2.2 Synergistic Damage Mechanisms of Neuroinflammation and Oxidative Stress

METH‐induced neurotransmitter dysfunction further triggers a synergistic cascade of neuroinflammation and oxidative stress, characterized by overactivation of microglia and astrocytes, and release of proinflammatory cytokines including TNF‐α and IL‐1β [29]. Gut microbiota dysbiosis is the key peripheral driver of this pathological cascade: Reduced microbiota‐derived SCFAs and increased lipopolysaccharide (LPS) from leaky gut activate the TLR4/NF‐κB pathway to amplify central neuroinflammation [25], while microbiota‐derived indole derivatives restore microglial homeostasis via AhR signalling, directly mitigating METH‐induced oxidative stress and inflammatory damage [26].

This neuroinflammation and oxidative stress form a robust positive feedback loop: reactive oxygen species (ROS) promote glial cell activation, while neuroinflammation further expands oxidative damage [30]. Activation of the NLRP3 inflammasome is closely linked to both processes and is modulated by gut microbial metabolites via peripheral‐to‐central inflammatory signalling [31]. Ultimately, the synergistic damage leads to neuronal apoptosis, synaptic dysfunction [32], blood–brain barrier (BBB) disruption [33] and METH‐induced affective and cognitive abnormalities, laying a pathological foundation for subsequent neural circuit dysfunction. Notably, the specific molecular mechanisms by which microbial metabolites precisely modulate the cross‐talk between neuroinflammation and oxidative stress in METH addiction have not been fully elucidated, and direct causal evidence from human clinical studies remains lacking.

## 3 Gut Microbiota Modulation of METH Addiction–Related Neural Circuit

METH‐induced molecular and inflammatory abnormalities are translated into addictive behaviours via dysfunction of reward, memory and impulse control neural circuits. Gut microbiota and its metabolites are critical peripheral modulators of these circuit functions, linking intestinal dysbiosis to aberrant circuit activity and addictive phenotypes via the microbiota–gut–brain axis [34, 35]. The core METH addiction–related circuits involve the ventral tegmental area–nucleus accumbens (VTA‐NAc) reward pathway and prefrontal–hippocampal networks, and gut microbiota modulate these circuits through well‐defined gut–brain axis mechanisms.

The specific regulatory evidence is as follows: (1) Microbiota‐derived SCFAs ameliorate METH‐induced anxiety‐ and depression‐like behaviours and suppress colonic inflammation, with effects dependent on the hippocampal SIGMAR1/BDNF/TrkB pathway [25]; (2) antibiotic‐induced gut microbiota depletion elevates circulating LPS levels, alters microglial morphology in the NAc and concurrently prevents the incubation of METH craving [34]; (3) FMT from METH‐exposed donors transfers anxiety‐ and depression‐like phenotypes to recipient mice, while SCFAs supplementation reverses these behavioural deficits and restores gut homeostasis [25]; (4) subdiaphragmatic vagotomy abolishes microbiota‐mediated behavioural and metabolic effects, confirming the essential role of the vagal gut–brain neural circuit in microbiota‐dependent modulation of METH‐related circuit dysfunction [36, 37].

**FIGURE 2:**
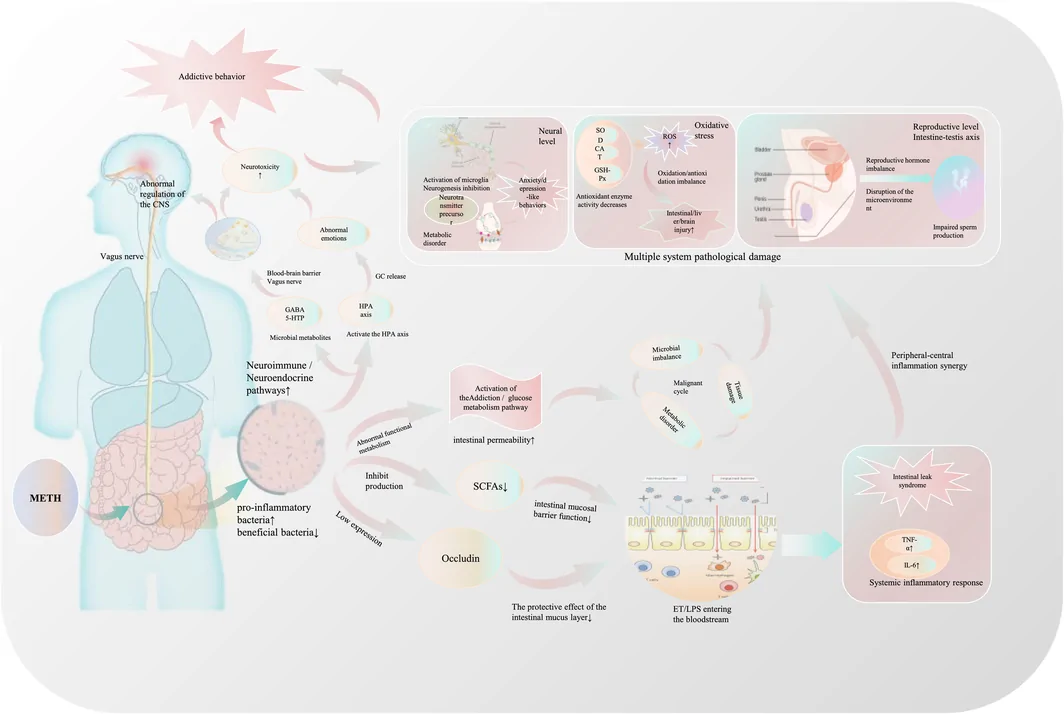
The regulatory mechanisms of METH on multiple systemic pathological damages. METH induces gut microbiota dysbiosis (increased proinflammatory bacteria and reduced beneficial bacteria), which further modulates multiple biological processes: it impairs intestinal mucosal barrier function (decreased SCFAs and Occludin, increased intestinal permeability), activates the HPA axis, affects the CNS via the vagus nerve, induces systemic oxidative stress, disturbs the intestine‐testis axis and causes leaky gut syndrome with endotoxin translocation. These processes interact to mediate neurotoxicity, addictive behaviours and extensive multisystem pathological damage.

These brain regions form a functional network through complex neural projections to jointly mediate core METH addiction behaviours. METH‐induced NLRP3 inflammasome activation, which is modulated by gut microbial metabolites, further drives long‐term circuit adaptation and increased drug‐seeking behaviour [38]. Clinical neuroimaging studies have identified abnormal functional connectivity patterns in MUD patients, which correlate with drug cue–induced craving and impulsivity [39, 40], but the causal link between these circuit abnormalities and gut microbiota dysbiosis in humans remains an unvalidated hypothesis, with no direct clinical evidence to date (Figure 2).

However, most existing studies examine adaptive changes in single brain regions individually and have not clarified how interregional abnormalities synergistically drive the dynamic progression of addictive behaviours via circuit linkage, nor have they fully elucidated the precise mechanisms by which microbial metabolites modulate circuit function across the BBB.

## 4 Gut Microbiota‐Mediated Epigenetic Regulation and Transgenerational Effects of METH Addiction

Epigenetic modifications drive the persistence of METH addiction phenotypes and transgenerational addiction susceptibility. Emerging evidence demonstrates that gut microbiota and its metabolites are key mediators of these epigenetic changes, linking intestinal dysbiosis to stable gene expression alterations in the CNS and germline.

Microbial metabolites directly regulate epigenetic modifications in the CNS and peripheral tissues: Microbiota‐derived SCFAs act as endogenous histone deacetylase (HDAC) inhibitors, modulating the expression of addiction‐related genes (including Fosb and dopamine system–related genes) in reward‐related brain regions such as the NAc, thereby regulating METH‐induced neuroplastic changes and addictive behaviours [41]. In addition, gut microbiota dysbiosis is associated with altered DNA methylation patterns in circulating leukocytes of METH users, which are linked to affective and behavioural abnormalities in clinical cohorts [42].

Paternal and prenatal METH exposure induces transgenerational enhancement of addiction susceptibility in offspring. Robust preclinical evidence shows that prenatal METH exposure causes persistent gut microbiota dysbiosis in offspring, which drives anxiety/depression‐like behaviours and altered reward sensitivity via the Wnt signalling pathway; prebiotic inulin supplementation reverses these phenotypes by reshaping the offspring gut microbiota [43]. It is hypothesized that microbiota dysbiosis may mediate transgenerational epigenetic effects via sperm‐borne noncoding RNAs, but direct causal evidence in METH addiction remains to be fully validated [44, 45]. Established studies have confirmed that parental addiction–induced histone modifications, DNA methylation and noncoding RNA alterations can transgenerationally affect offspring reward circuit function [46, 47], but the specific role of gut microbiota in mediating these transgenerational epigenetic changes has not been fully elucidated.

Notably, most current studies are limited to preclinical rodent models, and the causal link between gut microbiota, epigenetic modifications and METH addiction phenotypes in humans remains largely uncharacterized, requiring further verification in large‐scale clinical cohorts.

## 5 METH‐Induced Gut Microbiota Dysbiosis and Multisystem Pathological Cascade Reactions

The molecular, circuit and epigenetic pathological changes in the CNS that drive METH addiction do not occur in isolation. As the core functional hub of the microbiota–gut–brain axis, bidirectional crosstalk between gut microbiota and the CNS underpins the multisystem damage caused by METH addiction, forming a self‐reinforcing vicious cycle of ‘central pathological damage ‐ gut microbiota dysbiosis ‐ peripheral organ injury ‐ further deterioration of central function’.

METH exposure first triggers profound and consistent alterations in gut microbiota composition and metabolic function, directly disrupting intestinal barrier integrity. On a compositional level, METH exposure drives a marked depletion of beneficial commensal bacteria, including butyrate‐producing *Faecalibacterium* and *Lactobacillus* [43, 48, 49], alongside a significant enrichment of proinflammatory Proteobacteria, a pattern validated in both preclinical animal models and human MUD clinical cohorts [21, 22]. These compositional shifts drive widespread functional and metabolic disturbances, with the most prominent change being a significant reduction in the production of SCFAs including butyrate and propionate [37, 50]. As key mediators of intestinal homeostasis, SCFAs maintain intestinal mucosal barrier integrity, regulate mucosal immune responses and preserve tight junction structure. Reduced SCFAs directly impair intestinal barrier function, increase intestinal permeability and activate the proinflammatory TLR4/NF‐κB signalling pathway, driving the release of systemic proinflammatory cytokines including TNF‐α and IL‐6. Concurrently, METH‐induced microbiota dysbiosis downregulates the expression of tight junction proteins such as occludin [51]; combined with SCFAs deficiency‐related mucus layer impairment, this allows luminal endotoxins (e.g., LPS) to translocate into the systemic circulation, triggering a sustained systemic inflammatory response known as ‘leaky gut syndrome’. This peripheral inflammatory cascade forms a feedforward loop with central neuroinflammation, directly exacerbating core METH‐induced pathologies including neurotransmitter imbalance, microglia and astrocyte overactivation and synaptic dysfunction, which together establish the pathological basis for multiorgan damage in METH addiction.

Metagenomic analyses have further revealed that METH exposure reshapes the functional metabolic pathways of gut microbiota, amplifying systemic pathological damage [52]. Beyond inhibiting SCFAs biosynthesis, METH significantly disrupts the metabolism of aromatic amino acids including tryptophan and tyrosine, activates signalling pathways associated with substance dependence and alters glycolysis and glucose homeostasis, forming a vicious cycle of ‘microbial dysbiosis ‐ metabolic dysfunction ‐ tissue damage’ [52]. These microbial metabolic disturbances are the key mediators linking intestinal dysbiosis to central and peripheral pathology: Dysregulated tryptophan metabolism reduces the production of indole derivatives (key endogenous ligands for the AhR) [53], while altered tyrosine metabolism disrupts the precursor supply for dopamine synthesis, directly impacting the core reward pathways of addiction [35].

On this basis, METH‐induced gut microbiota dysbiosis drives multisystem pathological damage through multiple well‐defined pathways. At the neural and behavioural level, dysregulated microbial metabolism of neurotransmitter precursors (tryptophan, glutamate) directly disrupts central neurotransmitter balance via the microbiota–gut–brain axis [54]. Meanwhile, reduced microbial indole derivatives eliminate AhR‐mediated homeostatic regulation of hippocampal microglia, promoting proinflammatory M1 polarization of microglia, inhibiting hippocampal neurogenesis [26] and driving anxiety‐ and depressive‐like behaviours, as well as cognitive and reward circuit abnormalities that underpin addictive behaviours. At the reproductive system level, microbiota dysbiosis disrupts reproductive hormone balance and the testicular microenvironment via the gut–testis axis, impairing spermatogenesis and driving male reproductive toxicity [55, 56]. At the level of oxidative stress and inflammation, dysbiosis promotes intestinal ROS production [57, 58], reduces the activity of host antioxidant enzymes including superoxide dismutase (SOD) and disrupts the systemic oxidant‐antioxidant balance. This oxidative damage synergizes with endotoxin‐induced chronic inflammation to exacerbate injury to the intestine, liver and brain, further amplifying central oxidative stress and neuroinflammatory damage.

METH‐induced gut microbiota dysbiosis further amplifies central pathological damage and reinforces addictive phenotypes via neuroimmune and neuroendocrine pathways. On one hand, dysbiosis hyperactivates the HPA axis [59, 60], promoting glucocorticoid release, amplifying stress responses and anxiety‐like behaviours and forming a vicious cycle of ‘microbiota dysbiosis ‐ HPA axis hyperactivation ‐ affective disturbance ‐ increased drug craving and compulsive use’. On the other hand, microbial metabolites including GABA and 5‐hydroxytryptamine precursors regulate central neurotransmitter balance either via vagus nerve signalling or by directly crossing the BBB [1]. The critical role of this neural pathway is confirmed by studies showing that vagotomy blocks the regulatory effects of gut microbiota on METH‐induced neurotoxicity and behavioural abnormalities [37]. In addition, gut microbiota dysbiosis directly exacerbates the maintenance of METH addiction and relapse risk: Microbiota‐derived SCFAs regulate the expression of dopamine synthetase in key reward‐related brain regions including the NAc [1], modulating dopamine release and strengthening the rewarding effects of METH and drug craving. During METH withdrawal, microbiota dysbiosis inhibits BDNF/TrkB signalling [25, 34], impairing neuroplasticity, exacerbating the incubation of addiction memory and increasing relapse risk. These mechanisms are intertwined with the neuroinflammatory and oxidative stress cascade driven by dysbiosis, collectively driving the persistence and high relapse rate of METH addiction (Table 1).

**TABLE 1:** Gut microbiota dysbiosis associated with METH exposure.

Model	Design	Key findings	References
Pregnant mice	Prenatal METH exposure + cross‐fostering + inulin intervention	METH induces anxiety/depressive‐like behaviours in offspring, microbiota shifts from probiotic to proinflammatory; Inulin reshapes microbiota and activates Wnt pathway to reverse behavioural abnormalities	[43]
Mice	METH + antibiotics/FMT + SCFA or pioglitazone supplementation	Germ‐free or FMT proves microbiota is a key mediator of METH neurotoxicity and behavioural disorders; SCFA/Pio protect intestinal barrier and improve behaviour	[37]
Mice	METH‐CPP + *Lycium barbarum* polysaccharides	*Lycium barbarum* polysaccharides increase the abundance of probiotics like *Allobaculum* and *Gordonibacter*, attenuating addictive behaviour	[61]
Humans	78 MUD patients vs. 50 controls; 16S sequencing	Intestinal α‐diversity ↓, proteobacteria ↑; machine learning model with AUROC 0.906 distinguishes MUD	[21]
Humans (longitudinal)	62 METH users followed up during abstinence	Abstinence < 3 months: opportunistic pathogens ↑, probiotics ↓; prolonged abstinence: microbiota and impulsivity improve together	[22]
Mice	METH + SCFA administration + Sigmar‐1 KO	METH decreases SCFA, activates TLR4‐mediated enteritis; SCFA supplementation improves anxiety/depression via SIGMAR1‐BDNF/TRKB pathway	[25]
Mice	Chronic METH + antibiotics + FMT	Microbiota imbalance triggers microglial M1 polarization → hippocampal neurogenesis ↓ → spatial memory impairment; FMT is reversible	[62]
Rats	Intravenous self‐administration of METH + antibiotics + LPS	Germ‐free ↑ peripheral LPS, alters NAc microglial morphology and inhibits craving incubation; exogenous LPS simulates the same effect	[34]
Mice	METH + broad‐spectrum antibiotics → then FMT	Germ‐free blocks CPP, FMT restores it; FMT from healthy donors attenuates addictive preference	[63]
Mice	METH + Tlr4‐KO or antibiotics	METH induces liver damage and blood LPS ↑; Tlr4‐KO/germ‐free both attenuate liver inflammation and damage	[64]
Mice	Low‐dose chronic METH	Microbial diversity ↓, TLR4‐MyD88‐NFκB‐mediated colitis; serum sphingosine and 5‐HT imbalance associated with behaviour	[65]
Mice and Tlr4‐KO	METH disrupts intestinal barrier + promotes LPS leakage; Tlr4 deficiency or germ‐free reverses inflammation and barrier disruption	METH disrupts intestinal barrier and promotes LPS leakage; Tlr4 deficiency or germ‐free reverses inflammation and barrier disruption	[66]
Rats	14‐day METH → withdrawal	Both drug use and withdrawal alter microbiota structure; acute withdrawal induces depressive‐like behaviour, associated with microbiota changes	[67]
Rats	METH‐CPP receptor differences	High CPP positively correlates with *Akkermansia* abundance ↑; antibiotic pretreatment enhances addictive preference	[67]

## 6 Mechanisms of Targeting Gut Microbiota to Improve METH‐Related Pathological Symptoms

Given the core role of gut microbiota dysbiosis in the multisystem damage of METH addiction, regulating gut microbiota has become a potential target for improving METH‐related pathological symptoms. Its mechanism mainly focuses on reversing central–peripheral pathological pathways after restoring microbiota balance.

In terms of improving mental and addiction‐related behaviours, METH abuse is more likely to induce severe psychological disorders such as depression and anxiety than traditional drugs, and depressive symptoms often occur during withdrawal [68]. Gut microbiota plays a key role in withdrawal‐induced anxiety; eliminating gut microbiota with antibiotics or using germ‐free mice can prevent the occurrence of anxiety‐like behaviours; Gegen Qinlian Decoction (GQD) can significantly alleviate withdrawal‐related anxiety by balancing microbiota, improving intestinal permeability and reducing inflammation [69]. Supplementation of SCFAs can improve METH‐induced microbiota dysbiosis and colonic inflammation, and reduce depressive and anxiety‐like behaviours [25]; these symptoms are related to SCFAs produced by microbiota and the Sigma‐1 receptor (SIGMAR1) pathway, and SCFAs can alleviate symptoms by activating the SIGMAR1‐dependent BDNF/TRKB pathway; the therapeutic effect of metformin is related to microbiota metabolites (e.g., inosine) [48], which can also reverse anxiety and depressive‐like behaviours. In addition, gut microbiota is involved in the ‘incubation’ process of METH craving [37]; eliminating microbiota can block the morphological changes of microglia in the NAc core and prevent the development of craving behaviours. However, antibiotic‐mediated microbiota elimination or the use of germ‐free models affects the whole system; although they confirm the necessity of microbiota, they cannot distinguish whether these effects are caused by the absence of specific microbiota or changes in the overall microbial environment.

In terms of protecting against multisystem damage, METH‐induced male reproductive toxicity (decreased sperm quality, testicular lesions) is related to microbiota dysbiosis [41]; FMT experiments have confirmed that microbiota imbalance is a direct cause, and restoring microbiota balance can reversely improve reproductive system function; METH‐induced hepatotoxicity is also related to microbiota dysbiosis [49], and SCFAs supplementation may exert a protective effect by regulating metabolic pathways; meanwhile, METH‐induced microbiota dysbiosis, SCFAs reduction and intestinal barrier damage can be improved by FMT or antibiotic intervention to reduce intestinal permeability, alleviate leaky gut‐related inflammation and further reduce the impact of peripheral inflammation on the CNS; FMT and antibiotic intervention have also confirmed that gut microbiota regulation can improve METH‐induced neurotoxicity, motor disorders and cognitive deficits [37], and microbiota can affect vagus nerve signal transmission through the gut–brain axis.

The AhR also plays a key mediating role in METH‐induced neurobehavioral disorders, and this role is closely related to gut microbiota metabolism [26]: indole derivatives produced by gut microbiota through tryptophan metabolism can act as ligands for AhR. Supplementing withdrawal mice with indole derivatives and the AhR agonist Ficz can significantly restore microglial morphology and improve neurogenesis; studies using AhR knockout mice have found that the protective effect of indole derivatives on METH‐induced microglial dysfunction and neurogenesis defects is completely absent in AhR knockout mice. This confirms the specific role of indole derivatives in regulating microglial activity and neurogenesis through AhR and also highlights the core value of the gut microbiota—tryptophan metabolism—AhR pathway in improving METH‐related symptoms.

## 7 Gut Microbiota–Targeted Intervention Strategies for METH Addiction and Their Mechanisms

Based on the core regulatory mechanisms of gut microbiota in METH addiction detailed above, multiple microbiota‐targeted intervention strategies have been developed in preclinical studies and preliminary clinical trials. These strategies directly or indirectly modulate the composition and function of gut microbiota, reverse peripheral–central pathological disorders and provide novel directions for the treatment of MUD, with key preclinical and clinical evidence summarized in Table 2.

**TABLE 2:** Intervention strategies, outcomes, microbiota changes and mechanisms in METH research related to gut microbiota.

Intervention	Indicator	Microbiota	Mechanism	References
Probiotics	Significantly improved sleep quality, appetite and BMI.	Not reported	Alleviates intestinal microbiota dysbiosis–related symptoms, possibly indirectly affecting addiction‐related behaviours via overall health improvement.	[70]
Probiotics	Reduced METH‐induced impulsive behaviour.	Restored microbiota diversity decreased due to METH exposure.	Regulates intestinal microbiota structure to improve addiction‐related behavioural phenotypes.	[71]
Antibiotics/LPS	Antibiotic‐induced microbiota depletion significantly reduced METH craving behaviour; LPS supplementation also inhibited craving.	Broad‐spectrum antibiotics induced microbiota depletion.	LPS from enteric Gram‐negative bacteria activates microglia in the NAc, thereby reducing METH craving during withdrawal.	[34]
Antibiotics/FMT	Antibiotic treatment blocked METH‐CPP formation; FMT from healthy donors reduced CPP scores.	Antibiotics depleted microbiota; FMT restored microbiota.	Intestinal microbiota is a necessary condition for METH‐induced behavioural reward effects and neuroinflammation; reshaping healthy microbiota has therapeutic potential.	[63]
FMT	Mice receiving faecal transplants from METH users exhibited metabolic abnormalities related to METH pathophysiology.	Transplanted dysbiotic microbiota from human METH users to germ‐free mice.	Demonstrated that the dysbiotic microbiota in METH users itself is sufficient to mediate some pathophysiological phenotypes (e.g., metabolic abnormalities).	[72]
No intervention (observational)	METH users were associated with psychotic‐like symptoms.	α‐diversity (Shannon index) decreased; Bacteroidaceae and others decreased, Romboutsia and others increased.	METH abuse leads to intestinal microbiota dysbiosis, and microbiota changes are associated with tight junction protein downregulation and psychiatric symptoms.	[73]

### 7.1 Probiotics

Probiotics are live microorganisms that confer health benefits on the host when administered in adequate amounts. In a randomized clinical trial of MUD patients, probiotic supplementation significantly improved sleep quality, appetite, and BMI and reduced METH‐induced impulsive behaviours [25, 74], providing preliminary clinical evidence for their therapeutic potential. Preclinical studies further show that probiotics restore microbiota diversity, regulate gut–brain axis signalling and alleviate METH‐induced reward behaviours and neuroinflammation [75, 76].

In substance use disorders such as METH addiction, probiotics can regulate the bidirectional communication of the gut–brain axis, alleviate neuroimmune disorders and reward circuitry dysfunction and reduce drug craving and withdrawal symptoms. Their mechanism is related to promoting SCFAs production and regulating neurotransmitter generation (e.g., tryptophan metabolism) [77], which can improve emotional disorders and cognitive function; for example, in studies on autism spectrum disorder (ASD), probiotics can improve gastrointestinal symptoms and core behavioural problems [78], and this logic is also applicable to the field of addiction; METH addiction models also show that probiotics can reduce anxiety and depressive behaviours by restoring microbiota diversity [79]. Their potential effects include repairing microbiota dysbiosis, reducing systemic inflammation and decreasing relapse risk [80], making them a safe and easy‐to‐implement clinical intervention option.

### 7.2 Prebiotics

Prebiotics are indigestible dietary fibres that can selectively stimulate the growth and reproduction of beneficial bacteria (e.g., *Lactobacillus* and *Bifidobacterium*) in the intestine [81], indirectly regulating microbiota balance. In addiction treatment, prebiotics regulate gut–brain axis signal transmission by enhancing intestinal barrier function and increasing SCFAs levels, alleviating drug‐induced metabolic and neurobiological changes. Inulin is a common prebiotic; in the prenatal METH exposure model, supplementing inulin to maternal mice and their offspring can reshape microbiota composition [79], reduce hippocampal inflammation, promote neurogenesis and alleviate anxiety and depressive behaviours in offspring; prebiotics also play a key role in dietary regulation [82] and can support addiction treatment by regulating microbiota metabolic pathways. Their potential effects include restoring microbiota homeostasis, improving intestinal permeability and alleviating withdrawal symptoms (e.g., depression) [81], which are suitable for long‐term intervention, especially in combination with other strategies.

### 7.3 FMT

FMT involves transplanting faecal microbiota from healthy donors into the recipient's intestine to achieve comprehensive reconstruction of microbiota diversity, which is suitable for cases of severe microbiota dysbiosis. In METH addiction research, FMT is often used to verify the causal role of microbiota in addiction behaviours (e.g., depression, anxiety during withdrawal): Studies have shown that after METH withdrawal, mice that receive microbiota transplantation from addicted mice exhibit aggravated depressive and anxiety behaviors, accompanied by a decrease in the number of tryptophan metabolism‐related bacteria in the microbiota; in addition, FMT can also be used as a ‘rebalancing’ tool in substance use disorder treatment [83], repairing intestinal barrier damage and reducing the release of inflammatory factors. In addiction‐related neuroinflammation models, it can improve behavioural outcomes by regulating the gut–brain axis [82]. Its potential effects include rapidly resetting microbiota balance, which may serve as an alternative strategy for refractory addiction, but it requires individual adjustment to avoid adverse reactions and strict screening of donor microbiota to ensure safety.

### 7.4 Dietary Modulation

Dietary modulation directly affects microbiota composition and metabolites by adjusting dietary components (e.g., nutritional supplements or special dietary structures) [84, 85] and is the most accessible microbiota regulation method. In METH addiction, dietary modulation can correct microbiota dysbiosis and SCFAs reduction induced by drug exposure, alleviating gut–brain axis signal abnormalities. Omega‐3 polyunsaturated fatty acid supplements can reduce symptoms in high‐fat diet–induced obesity models by regulating body weight and microbiota composition [85], and this mechanism can be used in addiction treatment to improve metabolism and neuroinflammation; natural products from medicinal plants (e.g., long‐chain polysaccharides) can selectively regulate microbiota [86]; for example, low‐molecular‐weight polysaccharides are more easily fermented by microbiota, enabling efficient regulation of microbiota structure; in prenatal METH exposure experiments, dietary intervention (e.g., inulin supplementation) has shown potential in alleviating behavioural deficits [79], providing experimental basis for clinical dietary guidance.

### 7.5 Engineered Microbiota and Synthetic Biology Approaches

These approaches involve genetically engineering bacteria or bacteriophages to precisely regulate microbiota functions or metabolic pathways [3], and are precise intervention strategies targeting specific pathological mechanisms. In the field of addiction, engineered microbiota mainly target key pathways such as tryptophan and tyrosine metabolism, and alleviate drug craving and brain abnormalities by directionally expressing specific enzymes or metabolites. For example, engineered bacteria can be designed to deliver metabolites such as indole derivatives or related enzymes [83], restoring tryptophan homeostasis and reducing inflammatory responses. However, whether engineered bacteria can stably colonize, accurately express functions and not interfere with the host's native microbiota in the complex intestinal microenvironment is a core challenge for their translation from the laboratory to the clinic; in food addiction research, intestinal bacteriophages regulate addictive behaviours by modulating tryptophan and tyrosine metabolism [3], providing a potential model for METH addiction; in addition, bacterial exosomes can target the gut–bone axis in osteoporosis models [87], showing the ability to precisely regulate microbiota, and this characteristic can also be used for neuroaxis regulation in addiction treatment. Their potential effects include providing personalized treatment plans, which are suitable for refractory symptoms caused by microbiota metabolic defects.

### 7.6 Antibiotics, Antifungals and Synbiotics

Antibiotics and antifungals indirectly regulate microbiota balance by eliminating harmful bacteria; synbiotics are combinations of probiotics and prebiotics, which can enhance the overall intervention effect. These strategies are mostly used as auxiliary intervention methods. In addiction treatment, they are mainly used to control microbiota‐related inflammation and neuroimmune disorders: synbiotics improve core symptoms in ASD models by long‐term microbiota regulation [78], suggesting their application potential in the field of addiction; antibiotics and antifungals can reduce intestinal opportunistic pathogens and improve intestinal health [78]; although they have not been directly used for METH addiction, they have been confirmed to alleviate symptoms through SCFAs and immune signals in inflammation‐related mental disorders such as depression. Their potential effects include rapid intervention in cases of severe microbiota dysbiosis, but they need to be used with caution to avoid damaging beneficial symbiotic microbiota and are especially not suitable for long‐term use alone.

Despite promising preclinical results, all microbiota‐targeted interventions face common translational challenges. First, preclinical findings in rodent models have poor reproducibility in human trials, due to significant differences in gut microbiota composition, METH exposure patterns and the complexity of human addiction (including psychosocial factors) between rodents and humans. Second, most interventions lack large‐scale, randomized, double‐blind, placebo‐controlled clinical trials in METH use disorder patients, with no clear clinical endpoints for addiction outcomes (e.g., relapse rate and abstinence duration). Third, METH use disorder is often accompanied by comorbidities (HIV infection, hepatitis, psychiatric disorders, malnutrition), which alter the gut microenvironment and may exacerbate the safety risks of microbiota interventions. Finally, the long‐term effects of these interventions on host metabolic, immune and neuropsychiatric homeostasis remain unclear, requiring long‐term follow‐up in clinical studies.

## 8 Summary

This review systematically synthesizes the regulatory mechanisms of METH addiction and the research progress of gut microbiota–targeted interventions. At the mechanistic level, we clarify that the core molecular and neural basis of METH addiction centers on neurotransmitter system imbalance, synergistic damage of neuroinflammation and oxidative stress and dysfunction of addiction‐related neural circuits. We further establish that gut microbiota dysbiosis is a key peripheral driver of these central pathological changes. METH exposure induces significant depletion of beneficial bacteria including *Faecalibacterium* and *Lactobacillus*, enrichment of proinflammatory Proteobacteria and concurrent dysregulation of key microbial metabolites including SCFAs, tryptophan derivatives and bile acids. This dysbiosis mediates bidirectional gut–CNS communication through neuroimmune, neuroendocrine (HPA axis) and vagal pathways, further exacerbating the aforementioned central pathological damage. In addition, gut microbiota–driven epigenetic modifications and transgenerational effects enhance the persistence and heritability of addictive phenotypes. At the intervention level, we confirm that microbiota‐targeted strategies including probiotics, prebiotics, FMT and dietary modulation can effectively alleviate METH‐induced anxiety and depression‐like affective abnormalities, multisystem damage including neurotoxicity and reproductive impairment, and relapse risk. These therapeutic effects are achieved via restoring gut microbial homeostasis, repairing intestinal barrier integrity and normalizing microbiota–gut–brain axis signalling.

The core innovations of this review lie in breaking through the previous limitation that METH addiction research mostly focused on the CNS, and systematically positioning the gut microbiota as a core peripheral regulatory node in METH addiction for the first time. We construct a multisystem pathological chain model of ‘METH exposure ‐ gut microbiota dysbiosis ‐ abnormal microbiota–gut–brain axis signaling ‐ exacerbation of central pathology’, filling the research gap in the role of gut microbiota in the peripheral regulatory mechanisms of METH addiction. Meanwhile, we integrate multidimensional mechanisms including molecular pathways, neural circuits, epigenetics and gut microbiota regulation, and clarify that gut microbiota not only affects the CNS through microbial metabolites but also participates in the maintenance and transmission of addictive phenotypes through epigenetic modifications and transgenerational effects, effectively expanding the research dimensions of METH addiction mechanisms. Furthermore, based on the core regulatory role of gut microbiota, we systematically verify the effectiveness of multiple targeted intervention strategies, providing a novel ‘peripheral modulation ‐ central improvement’ therapeutic concept for METH addiction treatment, and laying a theoretical foundation for the development of innovative combination therapies targeting the microbiota–gut–brain axis to break through the current dilemma of lacking effective pharmacotherapies for MUD.

## 9 Current Research Challenges and Future Prospects

Despite the promising potential of gut microbiota–targeted interventions for METH addiction, current research still faces three core bottlenecks, which also define the key directions for future exploration.

At the mechanistic level, the causal link between gut microbiota and METH addiction remains insufficiently elucidated. Most existing studies are limited to correlation analysis, with no clear verification of the causal pathways through which microbial metabolites regulate withdrawal‐related affective disorders, drug craving and core reward circuit dysfunction. The specific druggable molecular targets of microbiota–gut–brain axis regulation are yet to be identified, and the role of gut microbiota in mediating the transgenerational epigenetic effects of METH addiction remains largely hypothetical, with no direct causal evidence.

At the experimental design level, the clinical translatability of current findings is severely limited. Rodent models cannot fully recapitulate the complexity of human METH addiction, including long‐term intermittent drug exposure, high comorbidity and psychosocial influencing factors. Most studies are based on short‐term intervention and observation, lacking long‐term dynamic tracking of gut microbiota during chronic addiction and prolonged abstinence. There is also a lack of standardized intervention protocols and uniform analytical methods across studies, with insufficient integration of multiomics data, leading to poor reproducibility and comparability of results.

At the clinical translation level, microbiota‐targeted interventions face multiple core barriers. The high individual heterogeneity of gut microbiota in MUD patients makes the therapeutic effect of interventions difficult to predict and standardize. The high comorbidity of MUD with infectious diseases, psychiatric disorders and malnutrition not only reduces the colonization efficiency of microbial interventions, but also increases potential safety risks. In addition, existing microbiota interventions have not been designed in synergy with METH‐induced central neuroadaptive changes, resulting in limited long‐term efficacy in maintaining abstinence and reducing relapse.

Future research should focus on the following core directions to address the above bottlenecks: (1) Identify the causal pathways and key druggable targets of the microbiota–gut–brain axis in METH addiction using conditional gene knockout, germ‐free models, and multiomics integration; (2) Establish animal models that more closely mimic human METH addiction, and validate mechanisms and interventions in large‐scale, multicenter clinical cohorts of MUD patients; (3) Develop personalized microbiota intervention protocols, and combine microbiota‐targeted strategies with central‐targeted pharmacotherapy or psychosocial interventions to improve long‐term abstinence outcomes; (4) Elucidate the role of gut microbiota in the transgenerational inheritance of METH addiction to provide a basis for early prevention of addiction susceptibility in high‐risk populations.

## Author Contributions

Jia Li (corresponding author) and Yishuang Dai were responsible for funding acquisition. All authors participated in the conception, design and analysis of the study, and all read and approved the final manuscript.

## Funding

This work was financially supported by the Guizhou Provincial Science and Technology Program, Project No. Qiankehe Basic Research‐ZK[2024] General 384.

## Conflicts of Interest

The authors declare no conflicts of interest.

## Data Availability

No new data were generated or analysed in this study.
